# Dataset of the first de novo transcriptome assembly of the arillode of *Baccaurea motleyana*

**DOI:** 10.1016/j.dib.2018.12.031

**Published:** 2018-12-13

**Authors:** Deden Derajat Matra, Arya Widura Ritonga, Azis Natawijaya, Roedhy Poerwanto, Sobir, Winarso Drajad Widodo, Eiichi Inoue

**Affiliations:** a Department of Agronomy and Horticulture, Bogor Agricultural University, Indonesia; b Research and Development Division, Mekarsari Fruit Garden, Indonesia; c College of Agriculture, Ibaraki University, Japan

## Abstract

*Baccaurea motleyana* Müll. Arg. (rambai) is one of the underutilized fruit natives to Indonesia, Thailand, and Malaya Peninsula and it is mostly cultivated in Java island (Lim, 2012) [1]. The edible part of fruits is white and reddish arillodes in which having sweet to acid-sweet tastes. However, nucleotide as well as transcriptome information of this species is still scarce, no information has been deposited in GenBank. In this data article, we performed for the first time of de novo assembly of transcriptome using paired-end Illumina technology. The assembled contigs were constructed using Trinity and after filtering and clustering, produced 37,077 contigs. The contig ranged 201–4972 bp and N50 has 696 bp. The contig was annotated with several database such as SwissProt, TrEMBL, nr and nt NCBI databases. The raw reads were deposited in DDBJ with DRA numbers, DRA007358. The assembled contigs of transcriptome are deposited in the DDBJ TSA with accession number, IADP01000001–IADP01037077 and also can be accessed at http://rujakbase.id.

**Specifications table**

**Table:** 

Subject area	Agricultural and Biological Sciences
More specific subject area	Horticulture
Type of data	RNA sequencing Data
How data were acquired	Illumina HiSeq X ten
Data format	Raw Sequencing reads and assembled contigs
Experimental factors	RNA sequencing was performed by using Illumina X Ten
Experimental features	RNA Sequencing of arillode tissue with reddish color at ripening stage
Data source location	Cileungsi, Bogor, West Java, Indonesia (6°24′50.1′′S 106°59′05.7′′E)
Data accessibility	The raw data have been deposited in the DNA Data Bank of Japan (DDBJ) under the DRA accession number, DRA007358 and the assembled contigs of transcriptome have been deposited in the DDBJ TSA repository with accession number, IADP01000001-IADP01037077 and also can be accessed at http://rujakbase.id
Related research article	Lim T.K., *Baccaurea motleyana*. In: Edible Medicinal and Non-Medicinal Plants, Springer, Dordrecht, 2012.

**Value of the data**

• These data provide transcriptome for the first time of *Baccaurea motleyana* from arillode fruits.
• These data will be useful to obtain molecular markers of microsatellite and single nucleotide polymorphisms for breeding program in *B. motleyana* and the related-genus.
• These data also will be valuable for gene expression analysis using any treatments among the species and related-genus.

## 1 Data

In this data article, a de novo transcriptome assembly of *Baccaurea motleyana* (rambai) has been reported for the first time. The tissue was collected from arillode-reddish color of rambai, and the high quality of RNA was extracted for 150 bp paired-end sequencing technology of Illumina. The high quality of reads was obtained, and de novo assembly was performed using Trinity v.2.4.0 [2]. All statistics of reads and assembled sequence were determined (Table 1). The contigs were reconstructed using CAP3 [3] and CD-HIT-EST v.4.6.8 [4] to remove redundant contigs and then the contigs were filtering and clustering using Corset v.1.06 [5]. The contigs were annotated with several databases using the BLAST v.2.7.1+ program [6]. An overview of the transcriptome assembly of *B. motleyana* is presented in Table 2.

**Table 1:** Read and assembly statistics of rambai (*Baccaurea motleyana*) arillode.

Features	Number
Reads (bases)	60,245,320/9,036,798,000
Number and bases total (bp) of transcripts	53,219/26,754,820
Number and bases total (bp) of unigenes	40,966/19,489,602
Number and bases total (bp) of contigs	37,077/19,675,275
Length range, average, and N50 of transcripts (bp)	201–4972/502.73/654
Length range, average, and N50 of unigenes (bp)	201–4972/475.75/609
Length range, average, and N50 of contigs (bp)	201–4972/530.66/696

**Table 2:** Functional annotation of rambai (*Baccaurea motleyana*) contigs.

Database source	Number of contig (%)
Contig number	37,077
- Non-redundant protein (nr) NCBI	25,647 (69.17%)
- Non-redundant nucleotide (nt) NCBI	22,712 (61.26%)
- Swiss-Prot UniProt	17,316 (46.70%)
- TrEMBL UniProt	26,299 (70.93%)

## 2 Experimental design, materials, and methods

*B. motleyana* (rambai) cultivar. Merah (reddish arillode) were collected from Mekarsari Fruit Garden at ripening stage. The flesh arillode was used for RNA extraction. The total RNA was extracted using ISOLATE RNA (Bioline) following the protocol. The quality and quantity of DNA were checked by P360 Nanophotometer (Implen, München, Germany). The total RNA was subjected to preparation of a paired-end library for RNA sequencing using the Illumina Hiseq X Ten (BGI, Hongkong). After sequencing, the raw reads were filtered includes removing adaptor sequences, contamination and low-quality read from raw reads. The high quality of reads used to construct the transcriptome contigs using Trinity package with default parameters and minimum length of 200 bp. The assembled contigs were performed by CAP3 (-p 90), and CD-HIT-EST (-c 0.90 -M 0 -T 0) and clustering with Corset after filtering low expression reads below 1 CPM. Several databases such as nt and nr databases from NCBI and SwissProt and TrEMBL databases from UniProt were used to annotate the contigs using the BLAST program with the cut-off of 10^−5^.

## 3 Data accessibility

All raw data and sequences have been deposited to the DDBJ with accession number DRA007358 and assembled contigs have been deposited to the Transcriptome Shotgun Assembly (TSA) with accession number, IADP01000001–IADP01037077 and also can be downloaded at http://rujakbase.id/content/download.
